# Analysis of Twitter Users’ Sharing of Official New York Storm Response Messages

**DOI:** 10.2196/med20.3237

**Published:** 2014-03-20

**Authors:** Nicholas Genes, Michael Chary, Kevin Chason

**Affiliations:** ^1^Department of Emergency MedicineIcahn School of Medicine at Mount SinaiNew York, NYUnited States

**Keywords:** social media, disaster response, emergencies, public health, emergency management

## Abstract

**Background:**

Twitter is a social network where users read, send, and share snippets of text (“tweets”). Tweets can be disseminated through multiple means; on desktop computers, laptops, and mobile devices, over ethernet, Wi-Fi or cellular networks. This redundancy positions Twitter as a useful tool for disseminating information to the public during emergencies or disasters. Previous research on dissemination of information using Twitter has mostly investigated the characteristics of tweets that are most effective in raising consumer awareness about a new product or event. In particular, they describe characteristics that increase the chance the messages will be shared ("retweeted") by users. In comparison, little has been published on how information from municipal or state government agencies spreads on Twitter during emergency situations. Retweeting these messages is a way to enhance public awareness of potentially important instructions from public officials in a disaster.

**Objective:**

The aim of this study is to (1) describe the tweets of select New York State and New York City agencies by public officials surrounding two notable recent winter storms that required a large-scale emergency response, and (2) identify the characteristics of the tweets of public officials that were most disseminated (retweeted).

**Methods:**

For one week surrounding Superstorm Sandy (October 2012) and the winter blizzard Nemo (February 2013), we collected (1) tweets from the official accounts for six New York governmental agencies, and (2) all tweets containing the hashtags #sandy (or #nemo) and #nyc. From these data we calculated how many times a tweet was retweeted, controlling for differences in baseline activity in each account. We observed how many hashtags and links each tweet contained. We also calculated the lexical diversity of each tweet, a measure of the range of vocabulary used.

**Results:**

During the Sandy storm, 3242 shared (retweeted) messages from public officials were collected. The lexical diversity of official tweets was similar (2.25-2.49) and well below the average for non-official tweets mentioning #sandy and #nyc (3.82). Most official tweets were with substantial retweets including a link for further reading. Of the 448 tweets analyzed from six official city and state Twitter accounts from the Nemo blizzard, 271 were related to the storm, and 174 had actionable information for the public. Actionable storm messages were retweeted approximately 24x per message, compared to 31x per message for general storm information.

**Conclusions:**

During two weather emergencies, New York public officials were able to convey storm-related information that was shared widely beyond existing follower bases, potentially improving situational awareness and disaster response. Official Sandy tweets, characterized by a lower lexical diversity score than other city- and Sandy-related tweets, were likely easier to understand, and often linked to further information and resources. Actionable information in the Nemo blizzard, such as specific instructions and cancellation notices, was not shared as often as more general warnings and “fun facts,” suggesting agencies mix important instructions with more general news and trivia, as a way of reaching the broadest audience during a disaster.

## Introduction

Social media platforms such as Twitter have proven useful for the rapid dissemination of information during and after disasters. Twitter, a service where users can share short messages of text with or without photos or links to websites, is resilient [1], available via cellular, Wi-Fi, or broadband connections on mobile or desktop computers. The messages have a global reach, but can be directed very locally.

Twitter has become a prominent way to rapidly disseminate information during and after disasters. In the aftermath of the 2010 Haiti earthquake [2] and 2011 Japan earthquake [3], local officials, survivors, and relief workers used Twitter to (1) communicate about available shelters and supplies, (2) co-ordinate search efforts to locate the missing, and (3) co-ordinate relief efforts such as raising money.

While governments and aid agencies have employed Twitter for constructive ends during emergencies, the first step in evaluating a public health intervention is assessing reach [4]. And yet, the characteristics of these Twitter messages during times of disaster remain unstudied.

Local health departments and public agencies routinely use Twitter to engage and educate the public [5]. Twitter could be useful in disasters, such as extreme weather events, when change communication management is imperative [6]. The coordination of messaging content amongst all response partners and affected individuals is a critical function in management of disasters.  Public information officers representing response agencies coordinate via Joint Information Centers to ensure coordinated public messaging. Twitter could be an important means to disseminate information during a disaster because it leverages existing social networks. Tweets can easily propagate to a wider audience when users “retweet” them, share the tweet with an audience that follows the retweeter. The original source, in this case the public Twitter account, can choose to allow or disallow retweeting.  Tweets can also be found if marked with “hashtags”, keywords preceded by “#”. Marking tweets up with hashtags organizes tweets around topics. Tweets marked with hashtags can easily be found with Twitter’s built-in search function. 

Twitter users routinely use hashtags to expand the reach of their messages, whether for typical use or in times of emergency. The study of maximizing retweets has been left to marketers and advertising-focused firms [7]. The characteristics of messages that increase their chance of being retweeted during disasters remain unstudied.

This is regrettable for two reasons. First, from the perspective of the public official in a time of emergency, there is enormous potential benefit to crafting a pithy message that is widely shared and seen by millions, with comparatively little extra effort to learn what would make a compelling message, and no extra cost.

Second, the need for accurate information from public officials in times of disasters is acute. In fact, inaccurate unofficial messages have been noted to proliferate quickly in times of disaster, as was the case after the Boston Marathon bombings [8]. Some public officials in Haiti viewed Twitter with suspicion after the earthquake, citing the rapid spread of rumors through backchannels [2]. It would be unfortunate if this useful tool was neglected by distrustful officials, instead of studied to better enhance its utility.

Recently, the New York area has seen a major weather event, Superstorm Sandy, as well as a significant snowstorm (dubbed “Nemo” by a weather channel [9]). To observe how public officials used social media during extreme weather events, we collected tweets from several public officials’ and agencies’ accounts on Twitter before, during, and after the storms hit. It was our objective to determine characteristics that led to increased sharing among message recipients, with the goal of improving future messaging during disasters.

## Methods

Inclusion criteria: for each event, we included tweets that (1) were pushed to Twitter in the week surrounding the event by selected official accounts, and (2) contained certain keywords.  We collected data from six official Twitter accounts: @311NYC, @NotifyNYC, @NYCGov, @NYCMayorsOffice, @NYGovCuomo, and @MikeBloomberg. We collected tweets from these accounts for a one-week period surrounding each extreme weather event; October 27 to November 2, 2012 for Superstorm Sandy, and February 5-12, 2013 for blizzard Nemo.

Although the inclusion criteria were the same for both events, we used different but comparable methods to acquire data surrounding each event. For the Sandy storm we collected tweets using custom software. For the Nemo blizzard, we collected tweets using cached version of the search results page of Twitter.com. The main difference between the two methods is that the custom software also provided tweets from non-official sources, which provided a frame of reference.

For Superstorm Sandy, we collected tweets that contained “#Sandy”  and “#NYC”  using custom software written in Python to acquire tweets from Twitter’s ReST API v1.0. For Nemo, we scraped each of the six official accounts. Scraping refers to extracting parts of a webpage when the HTML code representing the webpage is viewed in a text editor. 

Once the tweets were acquired, we identified which messages were related to the storm. We then identified which messages related to the storm had actionable information for the public. Retweets from various accounts were normalized to number of tweets and follower counts. Additionally, the most shared tweets were analyzed and compared to other public official tweets during the storm period.

The software is available at GitHub. Twitter’s terms of use prevents the redistribution of tweets, even for academic purposes. Those terms do allow the redistribution of identification numbers for each tweet, which we will provide on request.

## Results

### Superstorm Sandy

**Figure 1:**
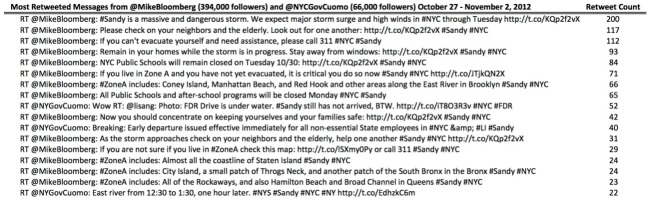
The 17 most-shared official messages during the Sandy storm.

We collected 50,014 tweets from the six public official accounts during the specified data range. Of those, 3242 tweets were retweeted. On three occasions, New York city mayor Michael Bloomberg and staff, tweeting from @MikeBloomberg, had tweets retweeted over 100 times. New York governor Andrew Cuomo and staff, tweeting from @NYGovCuomo, had several tweets retweeted 22-52 times during the analyzed period (Figure 1). @MikeBloomberg had the most followers of all analyzed accounts at the time (approximately 394,000 followers).

The lexical diversity of these official tweets was similar (2.25-2.49) and well below the average for non-official tweets mentioning #sandy and #nyc (3.82). Ten of the 17 official tweets with more than 20 retweets including a URL for further reading.

### Nemo Blizzard

**Figure 2:**
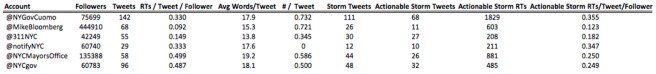
Retweet characteristics from six official accounts during the week of the Nemo blizzard.

We collected 448 tweets from the official accounts. Of these, 271 were related to the storm and 174 had actionable information for the public, such as train and school cancellations, or instructions for managing power outages. Actionable storm messages were retweeted on average 24 times. Messages with general storm information were retweeted 31 times. Figure 2 describes the retweet rates for each official source.****

**Figure 3:**
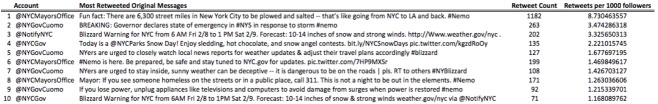
Top ten shared original storm-related official messages during the week of the Nemo blizzard.

Of the 10 most retweeted messages (an average of 255 retweets per tweet) for blizzard Nemo, 7 used hashtags, 5 had actionable information, and 4 had links or mentions to other official accounts for further reading (Figure 3).

For comparison, in the general population of retweets, 56% had hashtags, 64% contained actionable information, and 62% contained links for further reading. These most shared messages averaged 20.9 words per message, significantly more than the 17.2 words averaged other official tweets that week (student’s *t* test, *P*<.01).

## Discussion

### Principal Findings

This paper provides the first description of how the spread of information from official sources during an emergency relates to the structure of those messages.

Our study provides evidence that during emergencies the tweets from official sources that reach the widest public audience are those that are simple and self-contained.  The most retweeted official tweets had lower lexical diversity (simpler vocabulary), were longer than average, and contained no more hyperlinks than average.

Tweets from official sources to the general public may be more useful in establishing an official presence during an incident than in raising situational awareness or coordinating responses. The most retweeted tweets contained general tips or photos rather than actionable information.

Our data suggests that during emergencies official tweets reached a wider audience, which may have improved situational awareness and disaster response. The official tweets that were retweeted the most (had the widest reach) used simpler wording and were longer than average. This suggests that the most retweeted messages were those that were the simplest to understand.

### Comparison With Prior Work

Earlier work on Twitter’s retweeting suggested tweet is more likely to be retweeted if it originates from a user with a high number of followers, who also follows many other users and contains many URLs and hashtags [10]. A tweet is unlikely to be passed along (ie, a retweet of a retweet) more than 10 times [11]. A user’s tweet is more likely to be retweeted if that user has had prior tweets retweeted [12].  Zhu et al [13] found that more than 50% of a tweet’s retweets occur within the first hour after the original tweet is posted.

Other studies found that including a link increases the likelihood that a tweet will be retweeted [7]. Our study found that including links had no such effect.

Our study used retweets to quantify the degree of dissemination (spread) of information throughout a social network. We did not distinguish whether a tweet was a retweet or a retweet of a retweeted tweet, and so forth. Perhaps looking at the depth of retweets is a more accurate measure of the spread of information.

### Limitations

#### Methodological

Our analyses may be incompatible because we collected the data for each extreme weather event with different methods. The most substantial difference of this limitation is the lack of a reference population for the tweets concerning Nemo.

It is possible that, because of power outages, fewer people were using Twitter on their computer or conserving battery on their laptop or phone or tablet. An analysis of social media during the Sandy storm suggested that social media was only an adjunct to traditional media [14]. Thus, the Twitter activity seen during storms may disproportionately represent activity outside the New York area, bystanders who were not the intended target of the messages. This may explain why general tips and “fun facts” were shared more often than actionable information. We did not control for the location of retweeters in this analysis.

#### Data Formatting

Our study focused on retweets. How Twitter indicates that a tweet is retweeted varies with different platforms. The official method, introduced in 2009, is clicking a “retweet” option on a Twitter client. An older method involves cutting and pasting a tweet into a new tweet and pre-pending “RT” to it. Both methods are in use to varying extents across personal computers and mobile devices. The data concerning superstorm Sandy recognized both methods. The data concerning the Nemo blizzard only recognized the newer method.

### Conclusions

One reason for considering social media as part of an official emergency response plan is to rapidly disseminate accurate, up-to-date information to the public during what is typically a rapidly changing cycle of incident assessment and information dissemination to build presence and situational awareness. This study is an important first step in determining how municipal and state agencies can use social media to enhance emergency preparedness and response. Future studies can standardize the methods, control for additional variables such as location, and study a wider variety of disasters and emergency response systems.

## Abbreviations

API: application programming interface
RE-AIM: Reach, Efficacy, Adoption, Implementation, Maintenance
REST: representational state transfer
RT: retweet
